# Comparative Assessment of the Acute Effects of Whey, Rice and Potato Protein Isolate Intake on Markers of Glycaemic Regulation and Appetite in Healthy Males Using a Randomised Study Design

**DOI:** 10.3390/nu13072157

**Published:** 2021-06-23

**Authors:** Helena Tiekou Lorinczova, Sanjoy Deb, Gulshanara Begum, Derek Renshaw, Mohammed Gulrez Zariwala

**Affiliations:** 1 Centre for Nutraceuticals, School of Life Sciences, University of Westminster, 115 New Cavendish Street, London W1W 6UW, UK; w1505041@my.westminster.ac.uk (H.T.L.); S.Deb@westminster.ac.uk (S.D.); begumru@westminster.ac.uk (G.B.); 2 Centre for Sport, Exercise and Life Sciences, Institute of Health & Wellbeing, Coventry University, Priory Street, Coventry CV1 5FB, UK; derek.renshaw@coventry.ac.uk

**Keywords:** protein, animal-derived proteins, plant-derived proteins, whey protein, potato protein, rice protein, insulin, GLP-1, appetite, glucose homeostasis, type 2 diabetes mellitus (T2DM)

## Abstract

Global protein consumption has been increasing for decades due to changes in demographics and consumer shifts towards higher protein intake to gain health benefits in performance nutrition and appetite regulation. Plant-derived proteins may provide a more environmentally sustainable alternative to animal-derived proteins. This study, therefore, aimed to investigate, for the first time, the acute effects on glycaemic indices, gut hormones, and subjective appetite ratings of two high-quality, plant-derived protein isolates (potato and rice), in comparison to a whey protein isolate in a single-blind, triple-crossover design study with nine male participants (30.8 ± 9.3 yrs). Following a 12 h overnight fast, participants consumed an equal volume of the three isocaloric protein shakes on different days, with at least a one-week washout period. Glycaemic indices and gut hormones were measured at baseline, then at 30, 60, 120, 180 min at each visit. Subjective palatability and appetite ratings were measured using visual analogue scales (VAS) over the 3 h, at each visit. This data showed significant differences in insulin secretion with an increase in whey (+141.8 ± 35.1 pmol/L; *p* = 0.011) and rice (−64.4 ± 20.9 pmol/L; *p* = 0.046) at 30 min compared to potato protein. A significantly larger total incremental area under the curve (iAUC) was observed with whey versus potato and rice with *p* < 0.001 and *p* = 0.010, respectively. There was no significant difference observed in average appetite perception between the different proteins. In conclusion, this study suggests that both plant-derived proteins had a lower insulinaemic response and improved glucose maintenance compared to whey protein.

## 1. Introduction

Global protein consumption has been increasing steadily for decades [1] and is expected to continue to rise due to a combination of factors, which include changing socio-economic demographics [2] and changing consumer trends towards higher protein intake [3]. Health benefits frequently reported include improved free fat mass [4]; strength [5,6] and physical function [7] in adults. Furthermore, the satiating effects of protein have been well established [8,9,10]. In addition to reports describing a reduction in energy intake, protein consumption has the potential to impact weight loss [11]. The mechanisms responsible for protein-mediated appetite suppression include an alteration in gastric emptying [12] and modulation of regulation of gut-derived satiety hormones, including peptide YY (PYY), glucagon-like peptide-1 (GLP-1) and cholecystokinin (CCK) [13,14,15]. Furthermore, protein meals, and more prominently those containing whey protein, have been shown to have greater insulinaemic responses and improved regulation of postprandial glucose homeostasis [16]. Taken together, these observations suggest that dietary protein intake may have a positive association with metabolic and physical health.

Dairy foods are a rich source of proteins (e.g., whey and casein), which provide the necessary amino acids that the human body cannot synthesise [17]. Animal-derived proteins such as whey, are often cited as being of high quality due to their favourable rates of absorption, comprehensive amino acid profile and high levels of branched-chain amino acids (BCAA; leucine, isoleucine and valine); particularly leucine (~3 g/25 g whey protein), which stimulates muscle protein synthesis [18]. Whey and casein have also been shown to regulate appetite by increasing satiety and delaying the return of the feeling of hunger [19]. In addition, whey and casein have been reported to reduce subsequent energy intake [20,21], with an inclination towards whey having a superior effect [22]. These factors have led to an exponential growth in the adoption of whey protein in consumer products such as beverages and functional foods for appetite regulation and performance nutrition applications [23].

In parallel to the commercial growth of whey protein fortified functional foods and supplements, there has also been greater interest and adoption of plant-based eating patterns, such as vegetarian and veganism [24]. Various non-animal derived protein sources such as soy protein, rice protein and wheat protein have been explored scientifically and commercially in recent years, demonstrating varying levels of benefits and drawbacks [25,26]. Potato protein is a relatively novel source of plant-derived protein that provides a promising alternative to milk proteins [27]. Potato protein isolate is derived as a byproduct of starch manufacture and is therefore relatively cost-efficient compared to other protein sources. Furthermore, its nonallergenic, gluten and lactose-free characteristics make it an attractive dietary ingredient [28]. Interestingly, evidence from previous studies demonstrate that proteins from varied sources may differ significantly in their quality and consequently their satiating capacity [12,16,28]. It remains to be fully elucidated whether proteins from alternative sources can provide the identical metabolic benefits as those associated with milk proteins.

Towards this end, several assessments of protein quality have been put forward and various scales devised. Scales such as the protein digestibility–corrected amino acid score (PDCAAS), adopted by the World Health Organisation (WHO) [29] and the digestible Indispensable Amino Acid Score (DIAAS) [30] have been reviewed extensively elsewhere [30]. However, protein quality also needs to be balanced with effects on human health, at least in certain groups. Recent evidence from obese, insulin resistant groups indicate that the BCAA metabolite signature, indicative of increased catabolism of BCAA, were present and may be associated with the pathogenesis of obesity-associated insulin resistance [31]. In addition, the work of Rigamonti et al. [32] has implicated specific amino acids as having appetite suppressant and GLP-1 stimulating effects mediated via nutrient-sensing receptors in the gastrointestinal (GI) wall. Given the differing structural compositions and variable metabolic effects, it would be prudent to compare the appetite-regulating effects of proteins from differing sources.

To date, acute comparisons of appetite and glycaemic responses between animal and plant-derived protein have only focused on soy and pea protein isolates compared to whey [33,34,35]. These studies have suggested that soy and pea protein isolates elicit comparable effects on insulin, glucose and appetite regulation; however, to the authors’ knowledge, the evidence comparing plant-derived protein isolates, such as potato and rice protein, to whey protein appears to be sparse. The current study was designed to investigate the acute effects on glycaemic indices, gut hormones and subjective palatability and appetite ratings of two high-quality, plant-derived protein isolates; potato protein isolate (Solanic^®^100, ProteinmiXer.com^®^, Bonn, Germany) and rice protein isolate (Organic Oryzatein^®^ Silk 90, Axiom Foods/Growing Naturals, Inc., Los Angeles, CA, USA), against a high quality whey protein isolate-*Bi*PRO^®^ (Davisco Foods International, Inc., Eden Prairie, MN, USA) using a randomised, blinded cross-over design study. To the best knowledge of the authors, this is the first study of its kind that compares the above parameters between these protein isolates.

## 2. Materials and Methods

### 2.1. Participants

A total of 9 (from the randomized 12, Figure S1) male participants between the ages of 21 and 47 years completed the study. Participants’ suitability for the study was assessed using a Health Screening Questionnaire, completed on the first laboratory visit. Questions on pre-existing health conditions such as diabetes, high blood pressure and coronary heart disease (including whether on medication) were asked. In addition, dietary and supplementation aspects were captured. Individuals consuming more than 21 units of alcohol/week, having allergies to ingredients in the test shakes, suffering from illnesses that affect taste or appetite, gastrointestinal disorders, eating disorders, depression and/or smokers were not suitable for participation. Baseline anthropometric measurements were also collected on the same day by trained research staff. Height was measured using a Seca Leicester Height Measure (Seca GmbH & Co. KG, Hamburg, Germany). Weight, body mass index (BMI) and body fat % were measured using the Seca 515 medical Body Composition Analyser (Seca GmbH & Co. KG, Hamburg, Germany) and Bod Pod^®^ (Life Measurement, Concord, CA, USA). Participants signed written informed consent forms prior to participation. Ethical approval (ID: VRE1516-1375) was granted by the Faculty of Science and Technology Ethics Committee, University of Westminster, in accordance with the ethical standards of the Helsinki Declaration of 1975.

### 2.2. Study Design

**Figure 1:**
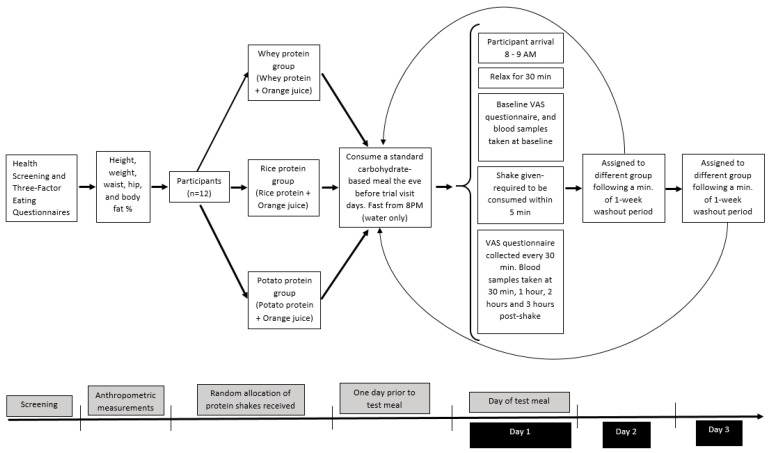
Overview of study design.

A single-blind (blinding of participants), randomised, triple cross-over study design was employed. Participants received three different protein shakes in a random order which was generated using the online service by Randomization.com [36]. Equal volume of the three isocaloric protein shakes prepared using whey, rice and potato protein powders were administered on different days, with at least a one-week washout period (Figure 1).

### 2.3. Study Protocol

Prior to the experimental trial days, the participants were instructed to attend the laboratory having abstained from caffeine intake and following a 12 h overnight fast. On arrival, a cannula was inserted in the antecubital fossa via an Introcan Safety^®^ IV catheter (20G) (B. Braun, Sheffield, UK), after which a baseline blood sample was collected (Time 0; T0). Participants were presented with one of the randomly assigned protein shakes (detailed below) from identical dark bottles and were instructed to drink the full amount within 5 min. Further blood samples were then drawn at timepoints T30, T60, T120 and T180 during each visit. Approximately 10 mL of blood was collected from each participant per timepoint using Becton Dickinson (BD) Vacutainer ^®^ EDTA tubes (BD, Oxford, UK). Blood in the EDTA (ethylenediaminetetraacetic acid) tubes was kept on ice and centrifuged (Hettich 340r, Hettich GmbH & Co. KG, Tuttlingen, Germany) within 2 h of collection, for 10 min at 3857 g. Plasma supernatants were aliquoted into 1.5 mL microcentrifuge tubes immediately post-centrifugation and stored at −80 ˚C. Furthermore, visual analogue scales (VAS) [37] were completed by all participants to measure subjective aspects such as palatability (measured during consumption of test shake) and satiety (measured at T0, T30, T60, T90, T120, T150 and T180).

### 2.4. Protein Shake Intervention

Participants were provided with the same volume of three different (whey, rice and potato) isocaloric protein shakes with an equal weight of protein content (Table 1). Protein content in each condition equated to approximately 45 g protein, as this had previously been shown to elicit satiating effects in a plant-derived protein (soy; [38]) and was above the threshold to elicit satiating effects from whey protein [39].

The shakes were prepared freshly on the visit day by mixing the required amount of the specific protein powder in 250 mL of liquid consisting of low sugar orange juice (Tropicana Trop50, Tropicana UK LTD, Leicester, UK) to improve palatability and water in predefined ratios. The orange juice allowed for the overall calorie and protein content to be matched across the three trials, to mitigate for their confounding effects on markers of appetite regulation. The composition of carbohydrates was within 2.7 g across the three conditions, which may result in a typical 0.5–0.8 mmol/L difference in blood glucose [40]. The three protein powders used were whey protein isolate (WPI; Instantized *Bi*PRO^®^, Davisco Foods International, Inc., Eden Prairie, MN, USA), rice protein isolate (RPI; Organic Oryzatein^®^ Silk 90, Axiom Foods/Growing Naturals, Inc., Los Angeles, CA, USA), and potato protein isolate (PPI; Solanic^®^100, ProteinmiXer.com^®^, Bonn, Germany). The WPI used was a dairy protein of high dispersibility [41] and high purity, with a 91% protein content (nutritional information provided by Davisco Foods International, Inc., Eden Prairie, MN, USA). The RPI used was sourced from whole rice grain, which is a suspendable food grade product with a protein content of 91% [42]. The PPI used was a product of protein absorption technology that enables the coagulation of protein from potato juice. The PPI is of high protein quality based on its DIAAS [27], with a protein content of 94% (nutritional information provided by Avebe, Veendam, The Netherlands) [27,43]. The full amino acid profiles of the protein powders are outlined in Table 2.

### 2.5. Plasma Analysis

Plasma was analysed to determine the primary outcomes of this study, which was to assess the acute effects of whey, rice and potato protein isolates on glucose, insulin, Homoeostasis Model Assessment–Estimated Insulin Resistance (HOMA–IR), total GLP-1 (amount of GLP-1, 7–36 and 9–36 forms), PYY and ghrelin. For each biomarker, assay kits were from the same Lot number and all samples were measured on the same day.

Plasma glucose concentrations were detected using a YSI 2300 STAT Plus Glucose Lactate Analyzer (YSI, Inc., Yellow Springs, OH, USA) and this is considered to be a gold standard method [44]. The YSI analyser utilises a steady-state measurement methodology based on the glucose oxidase technique (YSI STAT 2300 Plus laboratory manual). Plasma insulin concentration was analysed using an insulin ELISA (enzyme-linked immunosorbent assay) kit (DRG Insulin Elisa kit, DRG Instruments GmbH, Marburg, Germany). The measurement involved a solid phase enzyme-linked immunosorbent assay based on the sandwich principle. The analytical sensitivity was 0.076 ng/mL and the assay range was 0.076–4.33 ng/mL, and an intra-assay coefficient of variation (CV) of 2.5%, and inter-assay CV of 4.8% were determined. The measured plasma glucose and insulin levels were used to calculate HOMA–IR, a method for assessing β-cell function, using the following formula: fasting serum insulin (μU/mL) × fasting plasma glucose (mmol/L)/22.5 [45]. Total plasma GLP-1 was analysed using GLP-1 Total ELISA (Millipore Corporation, Billerica, MA, USA), a sandwich ELISA assay. The limit of sensitivity was 1.5 pM, and the assay range was 4.1 pM to 1000 pM GLP-1 Total/50 µl sample respectively, and an intra-assay CV of 2.1% and inter-assay CV of 9.8% were determined. Plasma peptide YY (PYY) was analysed using the Human PYY ELISA Kit (FineTest, Wuhan Fine Biological Technology Co., Ltd., Wuhan, Hubei, China). The kit’s sensitivity and detection range were 18.75 pg/mL and 31.25–2000 pg/mL, respectively, with an intra-assay CV of 6.6% and inter-assay CV of 9.4%. Total plasma ghrelin was detected using a competitive Millipore Human Ghrelin (Total) ELISA kit (Sigma-Aldrich, Darmstadt, Germany). The kit’s sensitivity was 9 pmol/L (20 µl sample size), with an intra-assay CV of 2.2% and inter-assay CV of 6.3%. All assay procedures were performed according to the manufacturer’s instructions and plates were read using a microplate reader (SPECTROstar^®^ Nano, BMG Labtech GmbH, Ortenberg, Germany).

### 2.6. Satiety and Palatability

The palatability of the protein shakes was assessed using VAS [37], consisting of five characteristics related to visual appeal, smell, taste, aftertaste and palatability (where 0 = good and 100 = bad). The satiety effects of the protein shakes were evaluated using a VAS measurement tool (a psychometric response scale using a 100 mm line with anchor statements at either end of the line). The satiety VAS contained eight characteristics of interest and included levels of hunger (0 = not hungry at all, 100 = never been hungrier), satisfaction (0 = completely empty, 100 = cannot eat another bite), fullness (0  =  not at all full, 100  =  totally full) and prospective food intake (0  =  nothing at all, 100  =  a lot). This data was used to calculate an average appetite response across the measurement period, using an adapted equation from Zafar et al. [46]:

Average appetite = [prospective food intake + hunger + (100 − fullness) + (100 − satisfaction)]/4

### 2.7. Statistical Analysis

The Shapiro–Wilk test provided no evidence to reject the hypothesis that all data were normally distributed. A two-way (condition (whey vs. potato vs. rice) * time) repeated measures analysis of variance (ANOVA) was used to assess differences in glucose, insulin, HOMA-IR, GLP-1, PYY, ghrelin, palatability, and average appetite. Furthermore, incremental area under the curve (iAUC) analysis was performed on glucose, insulin, GLP-1, PYY and ghrelin for each condition. Total iAUC was compared between whey, rice and potato for each variable using a one-way ANOVA. Where a significant main effect was found following the ANOVA, Bonferroni post hoc paired comparisons were performed. All analyses were carried out using IBM SPSS (v25.0 for Windows; SPSS, Chicago, IL, USA). The level of significance was set at *p* < 0.05. To establish statistical power, a post hoc power calculation was performed, with a reported power (1–β) greater than 0.8 for insulin, glucose, and GLP-1. In contrast, PYY and ghrelin had a power of 0.33 and 0.65, respectively. All descriptive data are presented as mean ± standard deviation unless otherwise stated.

## 3. Results

Nine healthy male participants volunteered for the study with the following characteristics (mean ± SD), age: 30.8 ± 9.3 years; height: 180.2 ± 7.5 cm; and bodyweight: 86.6 ± 6.7 kg.

### 3.1. Glycemic and Insulinaemic Response

**Figure 2:**
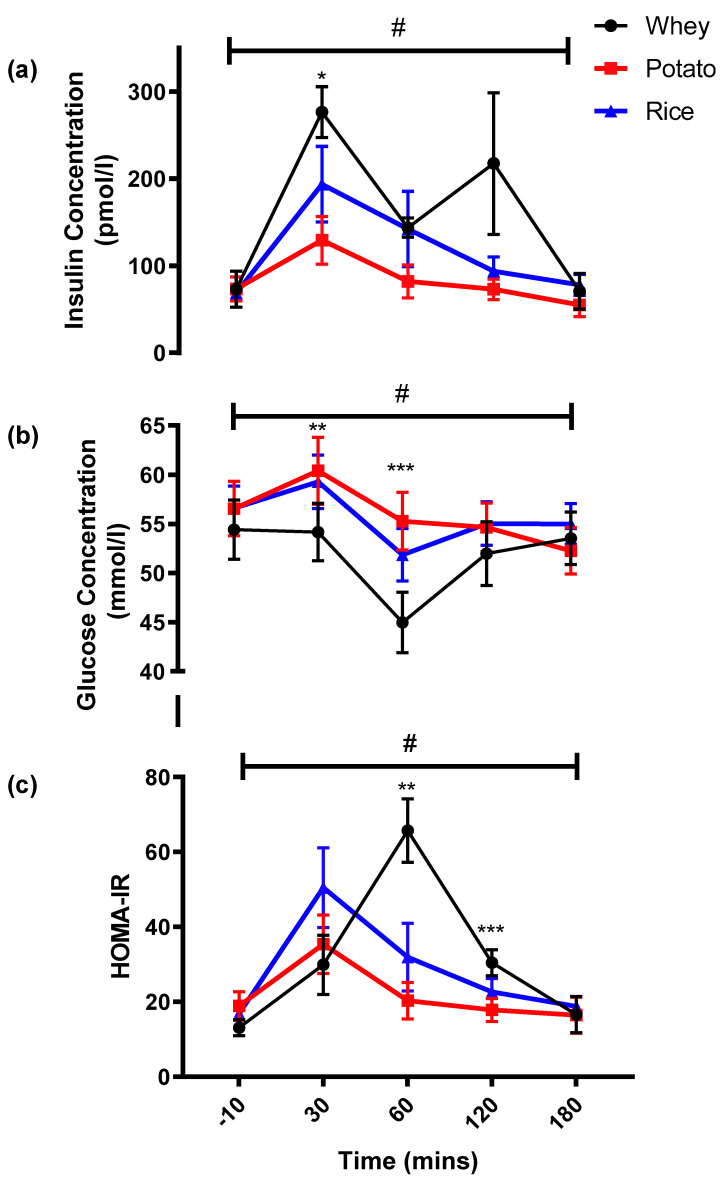
(**a**). Blood Insulin concentrations, (**b**) Blood Glucose concentrations and (**c**) HOMA-IR observed across the three experimental conditions. * Potato is significantly different to both conditions (*p* < 0.05). ** Whey is significantly different to both conditions (*p* < 0.05). *** Whey is significantly different from potato only (*p* < 0.05). # Shows that significance was present with time (*p* < 0.05).

There was an overall significant Condition * Time interaction for blood glucose (*p* < 0.001; Figure 2a). While these differences were not apparent at baseline, the whey condition had significantly lower blood glucose concentrations at 30 min compared to potato and rice conditions (*p* = 0.05 and *p* = 0.038, respectively), and this remained significantly different to the potato condition at 60 min (*p* = 0.004). No other differences were observed between conditions at other timepoints. Furthermore, time-related changes were also observed within all three conditions. In the whey condition, there was a significant reduction in glucose at 60 min to 4.5 ± 0.3 mmol/L compared to all other timepoints (all *p* < 0.05). A fall in glucose concentration was observed in the potato and rice conditions but this only reached significance at 120 min in the rice condition (vs. 30 min; *p* = 0.007); while the potato condition saw significant differences at 120 and 180 min (vs. baseline and 30 min; all *p* < 0.05). Overall differences in blood glucose were also observed with total iAUC comparisons (Table 3), with the whey condition showing significantly lower iAUC compared to potato (*p* = 0.048) but not rice (*p* = 0.082).

Blood insulin also displayed an overall significant Condition * Time interaction (*p* = 0.031; Figure 2b), with post hoc analysis revealing significant changes only occurred at 30 min as the potato condition remained significantly lower than the rice (−64.4 ± 20.9 pmol/L; *p* = 0.046) and whey (−141.8 ± 35.1 pmol/L; *p* = 0.011) conditions. Concerning time, differences were only observed in the whey condition as blood insulin peaked at 30 min, which was significantly greater than baseline, 60 and 180 min, but not 120 min. This was also reflected in the total iAUC being higher in the whey condition than potato (*p* = 0.013) and rice (*p* = 0.001).

The HOMA-IR also reflected the changes observed with glucose and insulin as a significant Condition * Time interaction was observed (*p* < 0.001; Figure 2c). The whey condition demonstrated a significantly greater response at 60 min compared to potato (*p* = 0.004) and rice (*p* = 0.009), but this difference was only sustained at 120 min with the potato condition (*p* = 0.024). From baseline, only rice saw a significant increase at 30 min (*p* = 0.026), but this fell towards baseline levels from 60 min onwards. In comparison, the whey condition saw a peak at 60 min (*p* < 0.05), which also remained significantly elevated compared to baseline at 120 min (*p* = 0.011). No differences were seen in the potato condition during the 180 min sampling period.

### 3.2. Appetite Related Hormones

**Figure 3:**
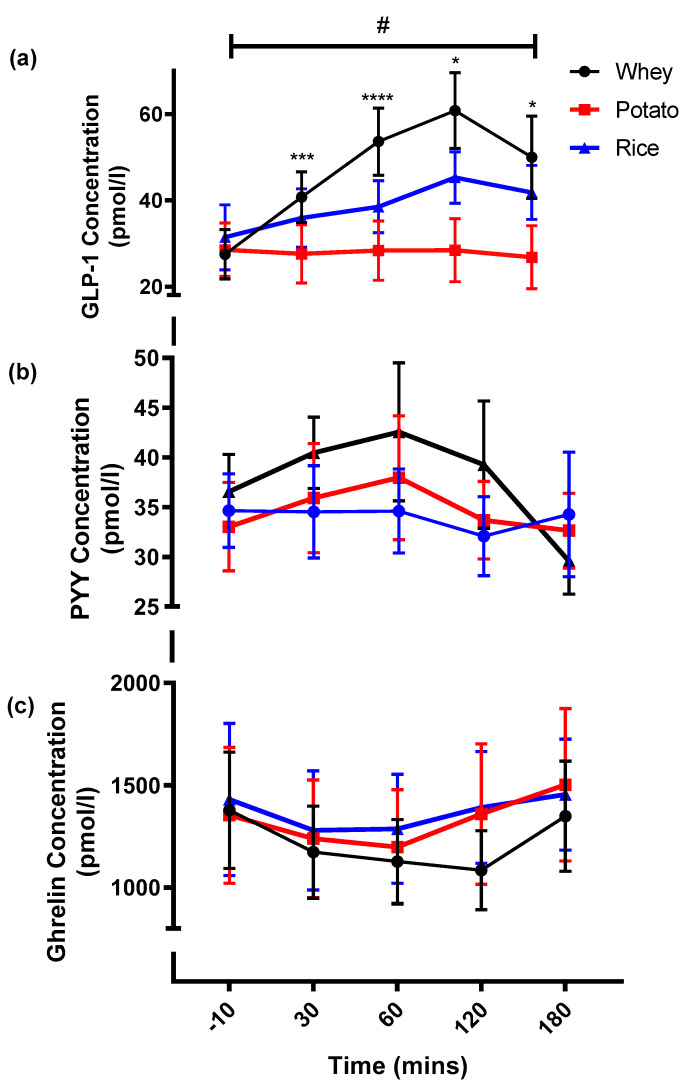
(**a**) Blood GLP-1 concentrations, (**b**) Blood PYY concentrations and (**c**) Blood ghrelin concentrations observed across the three experimental conditions. * Potato is significantly different to both conditions (*p* < 0.05). *** Whey is significantly different from potato only (*p* < 0.05). **** All conditions significantly different to each other (*p* < 0.05). # Shows that significance was present with time (*p* < 0.05).

Overall differences in GLP-1 were observed with a Condition * Time interaction (*p* <0.001; Figure 3a). The potato condition showed no change in GLP-1 throughout the measurement period and remained significantly lower compared to whey at 30 min (*p* = 0.007) and to both whey and rice at 60 min (*p* = 0.001 and *p* = 0.033, respectively), 120 min (*p* < 0.001 and *p* = 0.001, respectively), and 180 min (*p* = 0.007 and *p* = 0.001, respectively). While differences between whey and rice were present at 60 min (*p* = 0.001). The whey condition demonstrated an overall three-fold increase in concentration from baseline to 120 min (*p* = 0.001). Significant increases were present from baseline to 30 min (*p* = 0.001) and 60 min (*p* < 0.001), after which GLP1 concentrations began to plateau, as differences between 60 min and 120 min were not present (*p* = 1.0). The rice condition also exhibited a peak at 120 min, which was a significantly greater concentration than both the 30 min (*p* = 0.024) and 60 min (*p* = 0.04) timepoints. The iAUC calculations highlighted a significantly larger total iAUC in the whey condition compared to both the potato (*p* < 0.001) and rice conditions (*p* = 0.010; Table 3).

Differences in PYY were not detected by time or condition (Figure 3b), with any changes throughout the measurement period remaining nonsignificant. Equally, no differences in iAUC of PYY were calculated (*p* = 0.697). Ghrelin concentrations between conditions and time were also nonsignificant (Figure 3c; *p* = 0.290). However, there was a trend for greater total iAUC in the rice condition (Table 3), but this was also nonsignificant (*p* = 0.09).

### 3.3. Palatability and Satiety

**Figure 4:**
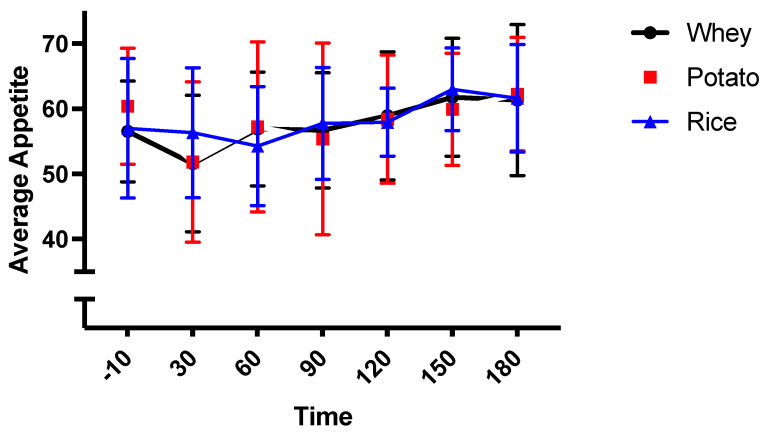
Shows average appetite perception across the three conditions.

The whey beverage was reported to possess greater visual appeal (*p* = 0.022) and palatability (*p* = 0.009) compared to the rice protein beverage. There were no other differences in smell, taste, aftertaste, visual appeal, or palatability observed between the three conditions (Table 4). Average appetite perception was not different between the protein conditions, but average appetite increased, showing a trend to significance with time (Figure 4; *p* = 0.013).

## 4. Discussion

This study demonstrated differing glycaemic and insulinaemic properties between potato, rice and whey proteins following ingestion. The insulin and glucose responses with whey protein were more substantial than both plant-derived proteins, with an acute rise in blood insulin accompanied by a reduction in blood glucose. Conversely, the glucose and insulin responses in rice and potato conditions were smaller, with a significantly lower response to blood insulin shown at 30 min following potato ingestion compared to whey. GLP-1 changes correspond to changes in insulin levels, with potato showing no change throughout the measurement period, but both rice and whey proteins increased with time to differing extents. However, appetite perception did not change as a result of the metabolic responses. Together, this suggests that characteristics of each protein, irrespective of plant or animal origin, may result in differing metabolic responses.

Potato protein isolate compares favourably with whey protein in terms of protein quality and has been scored 0.87 and 0.85 on the PDCAAS and DIAAS compared to whey protein, which scored as 1.0 and 0.90, respectively [3]. Rice protein fares less well in comparison to whey at 0.53 and 0.52, respectively [3]; although, comparisons of whey protein and brown rice protein supplementation on indices of body composition and exercise performance in resistance-trained males found no significant differences in post-exercise recovery and changes in body composition [47]. Significant absorption of total amino acids, essential amino acids, branch chain amino acids, and leucine have previously been demonstrated following a test meal of potato protein isolate in an acute feeding study [48]; although the appearance of amino acids in the blood following the meal was blunted and occurred later than whey protein. Proteins affect insulin secretion differently and may be influenced by the amino acid composition [49,50], but also by the rate of appearance of specific amino acids in the blood, including BCAA, phenylalanine, arginine and tyrosine [51,52,53], and/or the release of incretin hormones after ingestion of different proteins [52]. BCAAs, in particular, are thought to be potently insulinotropic [54,55]. In the current study, the total amount of BCAAs in the whey protein isolate and the potato protein isolate are well matched; however, given the results of previous studies [48], it is likely that the rate of digestion/absorption of whey protein derived BCAAs is more rapid than the plant-derived proteins and peaks earlier [48]. Nuttall et al. [56] previously demonstrated that incretin hormone release rather than total plasma amino acid levels is responsible for stimulating insulin release, which is in agreement with the current data. 

The stimulation and release of GLP-1 hormone by amino acids and dipeptides is triggered in the gut lumen on the apical surface of the entero-endocrine L cells rather than by plasma levels of absorbed amino acids [57]. L cells are located throughout the human GI tract [58], with the majority concentrated in the distal gut [59]. This luminal location of the GLP-1 sensing machinery may indicate that the rate of digestion and the appearance of specific amino acids may be key to triggering GLP-1 release rather than the rate of absorption per se. This more rapid rate of digestion of whey protein may be influential in stimulating incretin hormone release (in this case, GLP-1) in the current study. This data indicates that whey and rice protein isolates significantly increase levels of GLP-1 and insulin, whereas potato protein has no effect. 

Glutamine is a potent stimulator of GLP-1 release in endocrine L cell in vitro models, such as the GLUTag cell line [57]; however, this effect has not been demonstrated in vivo in humans. Interestingly, in the current study, glutamine levels were lowest in the potato protein isolate (7.9%; see Table 2) compared to whey (16.1%) or rice protein (17.9%). Therefore, the glutamine amino acid content of proteins may go some way to explaining the lack of GLP-1 hormone release in response to potato protein ingestion in the current study.

The current data, therefore, sheds more light onto the area of amino acid digestion/absorption and suggests that as well as the amino acid composition, the rate of digestion/absorption of BCAAs, essential amino acids (EAAs), total amino acids (TAAs) or individual amino acids may influence the release of incretin hormones (in this case GLP-1), which leads to insulinotropic effects. This may suggest that a threshold exists for incretin hormone release in the presence of amino acid digestion/absorption and that the potato protein described in the current study was below this threshold for GLP-1 sensing/stimulation. In contrast, whey and rice protein isolates breached this threshold and therefore triggered GLP-1 release, which stimulated insulin release to restore glucose homeostasis. Potato protein isolate also stimulates muscle protein synthesis during rest and following resistance exercise [60], indicating anabolic properties similar to whey protein.

Palatability and sensory characteristics are known drivers of food choice [61]. Although protein isolates are known to have a relatively better palatability profile compared to protein hydrolysates, very few studies have compared palatability between different proteins isolates in humans. This study shows that although whey protein demonstrated significantly greater visual appeal and palatability than rice protein, there were no other significant changes in smell, taste, aftertaste, visual appeal, and palatability between the three protein isolates. The variability in palatability between whey protein and rice protein also suggests that the use of low sugar orange juice as a flavouring agent did not fully mask the proteins’ original sensory and palatability characteristics.

## 5. Conclusions

This study is the first to observe the differing glycaemic, insulinaemic and appetite responses of potato and rice protein isolates compared to whey protein. While the metabolic effects of whey protein have been well established, the results demonstrate a dampened insulin response after the ingestion of the plant proteins, with potato protein maintaining better glycaemic regulation compared to whey. Equally, GLP-1 responses were also muted in the plant protein condition compared to whey, which included no changes in GLP-1 following potato protein ingestion. The differences in GLP-1 stimulation may be explained by the amino acid profiles of the protein isolates and, in particular, the lower concentrations of glutamine in the potato protein isolate. Taken together, this study sheds light on the implications of the protein source on glycaemic, insulinaemic and appetite regulation. These metabolic responses may elicit potential benefits for populations where tighter control of glycaemic and insulinaemic regulation may be beneficial while maintaining total protein intake.

## Figures and Tables

**Table 1:** Composition of protein shakes.

Protein Product	Product (g) in Shake	Protein (g) in Shake	Nutritional Composition of Orange Juice	Water (mL)	Total Energy Per Drink (Kcal)
Instantized *Bi*PRO^®^ (Whey Protein Isolate)	50	45.5	Volume: 207 mL Calories: 43.5 kcal Carbohydrates: 8.0 g Protein: 0.6 g	43	233.5
Oryzatein^TM^ 90 (Rice Protein Isolate)	50	45.5	Volume: 183 mL Calories: 43.5 kcal Carbohydrates: 7.1 g Protein: 0.5 g	67	233.4
Solanic^®^100 (Potato Protein Isolate)	48	45.3	Volume: 250 mL Calories: 52.4 kcal Carbohydrates: 9.8 g Protein: 0.75 g	0	233.5

**Table 2:** Typical amino acid profile expressed per 100 g protein.

Amino Acids (AA)	Whey Protein Isolate (%)	Rice Protein Concentrate (%)	Potato Protein Isolate (%)
Histidine *	2.0	2.2	1.8
Isoleucine *^,+^	5.6	4.2	5.9
Leucine *^,+^	12.7	8.3	9.8
Lysine *	10.2	2.9	7.6
Methionine *	2.3	3.0	1.1
Phenylalanine *	3.5	5.6	7.0
Threonine *	4.7	3.7	4.7
Tryptophan *	2.9	1.5	1.5
Valine *^,+^	5.4	5.6	8.6
Arginine	2.4	8.3	4.8
Cysteine	2.8	2.4	2.4
Glutamic Acid/Glutamine	16.1	17.9	7.9
Glycine	1.7	4.5	5.7
Proline	4.7	4.8	5.4
Tyrosine	3.6	5.6	5.4
Alanine	4.9	5.7	2.5
Aspartic Acid/Asparagine	11.4	8.9	13.1
Serine	3.3	5.1	4.8
**Total EAA**	49.3	37	48
**Total BCAA**	23.7	18.1	24.3

* Essential Amino Acid (EAA), ^+^ branch-chain amino acids (BCAA).

**Table 3:** Mean (±SD) total iAUC for blood glucose, plasma insulin, GLP-1, PYY and ghrelin.

	Whey	Potato	Rice	Overall Significance
Glucose	2.16 ± 3.8 *	14.01 ± 11.50	13.24 ± 12.3	0.013
Insulin	20,290.71 ± 14,222.9 #	3293.2 ± 2179.5	9002.8 ± 9564.8	<0.001
GLP-1	4248.5 ± 1323.1 #	325.1 ± 596.7	1646.4 ± 1289.1	<0.001
PYY	511.7 ± 930.1	794.7 ± 923.8	905.4 ± 1076.9	0.697
Ghrelin	1433.9 ± 22268.8	4668.3 ± 5540.3	17,186.1 ± 24,893.6	0.130

* Whey is statistically significant compared to Potato (*p* < 0.05); # Whey is statistically significant compared to Potato and Rice (*p* < 0.05).

**Table 4:** Mean (±SD) scores from the VAS questions on palatability.

	Whey	Potato	Rice	Overall Significance
Smell	27.9 ± 21.8	38.4 ± 30.4	36.7 ± 18.4	0.102
Taste	36.9 ± 26.6	52.3 ± 36.2	53.5 ± 22.0	0.173
Aftertaste	44.7 ± 32.7	56.6 ± 31.0	56.1 ± 31.0	0.06
Visual appeal	24.3 ± 23.0 *	37.3 ± 29.6	42.6 ± 27.4	0.022
Palatability	33.7 ± 21.9 *	54.1 ± 35.5	67.0 ± 27.7	0.009

* Whey is statistically significant compared to Rice only (*p* < 0.05).

## Data Availability

The data presented in this study are available on request from the corresponding author. The data are not publicly available due to ethical, legal and privacy issues.

## Supplementary Materials

The following are available online at https://www.mdpi.com/article/10.3390/nu13072157/s1, Figure S1: CONSORT flow diagram showing number of participants through each stage of the randomised cross-over trial.
