# Supplementary material for: Comparative Assessment of the Acute Effects of Whey, Rice and Potato Protein Isolate Intake on Markers of Glycaemic Regulation and Appetite in Healthy Males Using a Randomised Study Design
Source: Nutrients. 2021 Jun 23;13(7):2157. doi: 10.3390/nu13072157 (PMC8308460; doi:10.3390/nu13072157)

Figure S1: CONSORT flow diagram showing number of participants through each stage of the randomised cross-over trial.

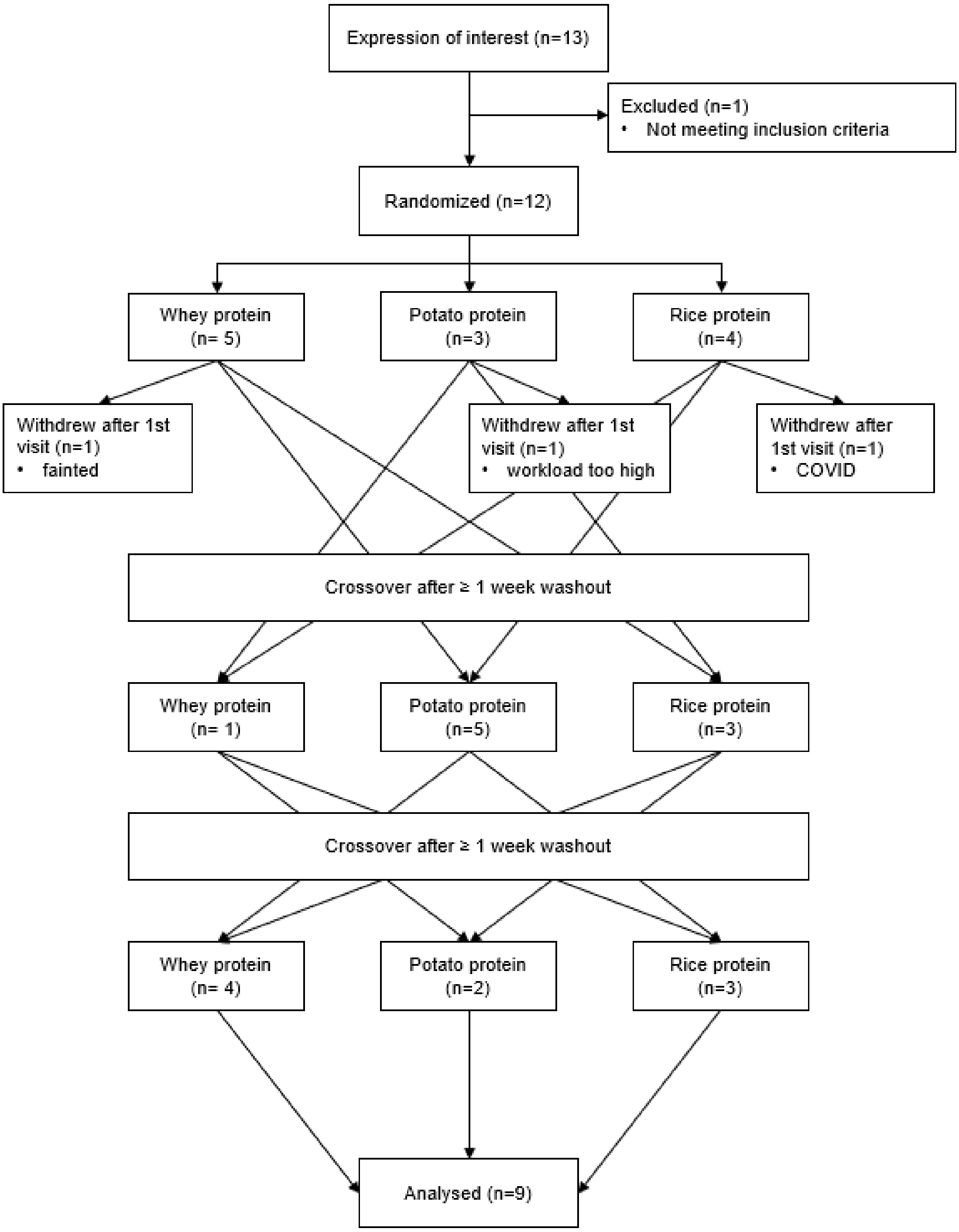

Supplement: Supplementary file 1 [file nutrients-13-02157-s001.zip › nutrients-1223300-supplementary.pdf]
